# Black Border Increases *Stomoxys calcitrans* Catch on White Sticky Traps

**DOI:** 10.3390/insects9010013

**Published:** 2018-02-02

**Authors:** Archie K. Murchie, Carol E. Hall, Alan W. Gordon, Sam Clawson

**Affiliations:** Sustainable Agri-Food Sciences Division, Agri-Food and Biosciences Institute, 18a Newforge Lane, Belfast BT9 5PX, Northern Ireland, UK; carol.hall@afbini.gov.uk (C.E.H.); alan.gordon@afbini.gov.uk (A.W.G.); sam.clawson@afbini.gov.uk (S.C.)

**Keywords:** stable fly, trap, contrast, farmyard, *Pollenia*, livestock

## Abstract

Stable fly, *Stomoxys calcitrans*, is a biting fly that can cause severe irritation to livestock resulting in reduced productivity. The most common method of monitoring *S. calcitrans* is through the use of sticky traps and many designs have been developed using different colours and materials such as alsynite fibreglass and polypropylene sheeting. Laboratory experiments and some field experimentation have demonstrated that colour contrast can attract *S. calcitrans*. However, this response has not been fully utilised in trap design. To test that simple colour contrast could increase trap efficacy, white sticky traps were mounted on three differently coloured backgrounds (white, yellow, and black) and positioned at five sites on a mixed livestock farm. White sticky traps on a black background caught significantly more *S. calcitrans* than the yellow or white backgrounds. An incidental result was that *Pollenia* sp. were caught in greater numbers on the yellow framed traps. The reasons for *S. calcitrans* attraction to black–white contrast are most likely due to conspicuousness in the environment although the extent to which flies are using this feature as a host-location cue or a perching site are unknown.

## 1. Introduction

Stable fly, *Stomoxys calcitrans* (L.) (Diptera: Muscidae), is a cosmopolitan haematophagous fly that frequently attacks livestock in farmyards. The three larval instars develop in decaying organic matter and adults blood-feed on a wide variety of mammalian hosts but predominantly cattle, horses, and dogs. Humans may also be bitten when there are insufficient numbers of suitable hosts in the vicinity. In Northern Ireland, there is anecdotal evidence to suggest that their populations are increasing; perhaps due to greater availability of breeding sites, such as discarded silage, feedstuffs, and soiled bedding, following intensification of agricultural practices. Both sexes of *S. calcitrans* blood-feed. High populations can increase cattle irritability, resulting in stamping, tail flicking, and head tossing, and stunting productivity. Consequently, *S. calcitrans* is considered the most important economic ectoparasitic pest of cattle in the US, with annual losses greater than $2 billion [1]. In addition to their nuisance impact, *S. calcitrans* is also a potential mechanical vector of equine infectious anaemia, lumpy skin disease, and others. They are an intermediate host for stomach worms (*Habronema* spp.) [2].

Sticky trapping of adult flies is common practice in North America to both monitor *S. calcitrans* prevalence and also to provide a level of control. Correspondingly, a number of trap designs have been developed. The standard trap is a cylinder of alsynite fibreglass surrounded by a clear sticky sleeve [3], commercially available from Olson Products Inc., Medina, OH, USA. The alsynite fibreglass reflects polarised light in a way that is particularly attractive to *S. calcitrans* [4,5]. Twin-walled polypropylene sheeting, often termed corrugated plastic (known by various trade-names e.g., Coroplast^®^ in North Armerica, Corriboard^®^ in UK/Ireland) has proven comparable to alsynite in catching *S. calcitrans* [6,7], although as with all visual traps this is dependent on colour. Cilek [7] caught significantly more *S. calcitrans* on blue boards compared to orange or white. However, Beresford and Sutcliffe [6] caught the greatest numbers of *S. calcitrans* on white traps, followed by grey, red, green, yellow, blue, and least on black—at c. 30% of the catch on white traps. The sex and physiological state of flies can also affect their responses to coloured traps. White traps caught proportionately more males than yellow traps [6]. Zhu et al. [8] found white traps caught more *S. calcitrans* in the field compared to blue-black patterned traps, although in a laboratory study, blue traps caught more gravid females, whilst white traps caught more young flies.

As with other haematophagous flies, colour contrast is also attractive to *S. calcitrans* [5,9,10,11]. Patterned inflatable beach balls painted with non-drying glue caught more *S. calcitrans* than their single-colour black or white counterparts [12]. Similarly, a combination of blue and alsyinite traps caught more *S. calcitrans* than the alsynite alone [13]. Using electrified targets, Schofield [14] found that blue-black patterns altered the numbers of *Stomoxys* spp. caught, with a vertical black stripe on a blue background resulting in the greatest catch. Brady and Shereni [15] assessed the landing responses of *S. calcitrans* to a variety of black and white patterns. They found that *S. calcitrans* were caught disproportionately more often on the region of interface between the two colours.

Despite clear indications that pattern and particularly contrast increase *S. calcitrans* catch, comparatively few modifications have been made to existing traps. The aim of this study was to determine if simple colour patterns could influence the numbers of *S. calcitrans* caught on sticky traps, with the specific hypothesis that greater colour contrast will result in more *S. calcitrans* caught.

## 2. Materials and Methods

### 2.1. Trap Design

**Figure 1:**
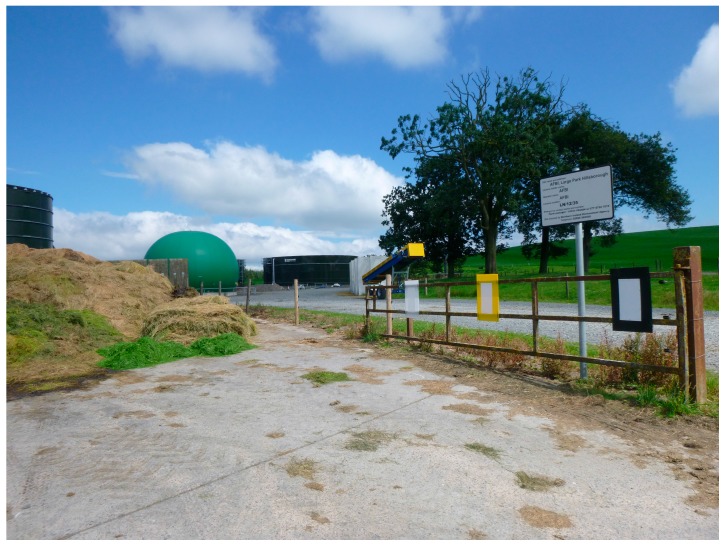
The basic experimental design, showing white sticky traps on white, yellow or black Corriboard^®^ frames mounted on pre-existing structures and positioned close to likely *S. calcitrans* habitats within a livestock-rearing farmyard. This is site 1 as described in the main text.

Traps consisted of white sticky traps 225 × 400 mm (Oecos, Kimpton, UK) attached to 4-mm thick twin-walled polypropylene sheet (Corriboard^®^, NI-Plastics, Downpatrick, Northern Ireland) measuring 425 × 600 mm. This resulted in a white central target (900 cm^2^) surrounded by a 100 mm-wide Corriboard^®^ frame. Three colours of Corriboard^®^ were used to provide the backdrop frame to the white sticky trap: translucent white, yellow and black (Figure 1). White polypropylene sheeting is attractive to *S. calcitrans* [6] and was viewed as the ‘standard’ or positive control treatment, black provides high contrast with the central white trap, and yellow is typically more attractive to phytophagous than haematophagous Diptera. Unfortunately, spectral reflectance data were not available.

### 2.2. Experimental Design

The experiment was conducted at the Agri-food and Biosciences Institute’s (AFBI) Hillsborough research farm, Co. Down, Northern Ireland (54.445290° N, 6.065526° W), which has on-site dairy, beef, sheep, and pig units. Traps were mounted in a portrait orientation, with the Corriboard® veins running vertically, on pre-existing structures (e.g., fences and gates) and close to livestock areas but out of reach of animals. Trap height was kept constant for all treatments at each site; although there was variation amongst sites, all traps were positioned with their bottom edges at 400–800 mm from the ground, with little vegetation in front of the traps, bearing in mind the recommendations of Beresford and Sutcliffe [16]. Five sites around the farm were chosen, three in the farmyard and two in close-by pasture. Site 1: beside the midden containing dung, waste feed and silage. Dairy cows passed this site on their way to the milking parlour. Site 2: at a walkway that dairy cattle used when moving to and from the dairy unit. Site 3: at a concrete collecting apron outside an occupied cattle shed. Site 4: on a roadway on the outskirts of the farm yard, with grazing cows, calves, and lambs in the adjoining paddocks. Site 5: in front of a forested area adjacent to fields with grazing cattle.

The experiment ran over six weeks from 24 July to 4 September 2017. One trap of each frame colour was mounted at each site. Traps were equidistantly spaced 1.8 m apart (at site 3, traps were 1.0 m apart due to space limitations). Traps were collected each week, changed, and the colour re-positioned according to a partially randomised design whereby each colour occupied each position twice during the six-week experiment. Treatments were therefore replicated at five sites, additionally each site was sampled on six occasions.

### 2.3. Data Analyses

*Stomoxys calcitrans* were counted directly on sticky traps. To sex *S. calcitrans*, they were picked off of traps, washed in petroleum ether, air-dried, and examined under a binocular microscope. Other muscid or calliphorid Diptera caught on traps in notable numbers were similarly treated.

Count data were subjected to generalized linear mixed models (GLMMs) fitted with Poisson distributions and logarithmic link functions. To examine whether treatments affected the sex ratio (proportion males) of *S. calcitrans* catches, a similar GLMM analysis was run but this time fitted to the binomial distribution with a logit link function. The significance of the fixed effects, frame colour, and site habitat in the models was assessed by comparing Wald statistics for each term against an F- or chi-squared distribution as appropriate. Site and position were fitted as random effects. All analyses were conducted using the statistical package GenStat v16.2 (VSN International Ltd., Hemel Hempstead, UK; www.vsni.co.uk).

## 3. Results

*Stomoxys calcitrans* catches were relatively low, with a maximum of 59 caught in any one trap per week. The three farmyard sites caught considerably more *S. calcitrans* than the two outlying pasture sites (Table 1; Wald statistic = 28.08, *df* = 1, *P* < 0.001). Across all sites, the black framed traps caught approximately five times more *S. calcitrans* than either the white or yellow framed traps (Table 1; Wald statistic = 270.20, *df* = 2, *P* < 0.001). There was no difference in the sex ratios of *S. calcitrans* caught on different colour framed traps (*F* statistic = 0.77, *df* = 2, 36, *P* = 0.471).

In the pasture sites, more cluster flies, *Pollenia* sp. (Diptera: Calliphoridae), were caught on the yellow-framed traps compared to the black or white framed ones (Table 1; Wald statistic = 61.14, *df* = 2, *P* < 0.001). Unfortunately, *Pollenia* sp. could not be identified to species level with certainty due to the condition of the flies after being removed from the sticky traps.

## 4. Discussion

A wide variety of traps have been developed to catch *S. calcitrans*. The traps used in this experiment have the advantage of being simple and cheap. They utilised twin-walled polypropylene sheeting (Corriboard^®^), which has been developed as a *Stomoxys* trapping material in the USA [6,7], coupled with commercially-available white sticky sheets. If double-sided white sticky traps are used, they are self-adhesive to the Corriboard^®^ making construction straightforward. A disadvantage of sticky traps that has gained some prominence in recent years is that they are used as a disposable item, rather than being re-usable, and are not recyclable. This is mostly a problem when large numbers of traps are used for control rather than for monitoring [17]. Nevertheless, it would be good to see traps constructed from biodegradable or otherwise recyclable material.

More *S. calcitrans* were caught in farmyard rather than nearby pasture sites. This reflects the proximity of the traps to *S. calcitrans* larval development habitats [18]. *Stomoxys calcitrans* larvae develop in decaying or faecally-soiled organic matter rather than pasture dung pats [19]. Overall though, the numbers of *S. calcitrans* caught were low compared to figures from other studies. In part, this is explained by the smaller trapping area, e.g., the sticky area in this study was 900 cm^2^, whereas the standard alsynite trap is a 30 cm high, 30 cm diameter cylinder [3] giving an area of 2826 cm^2^. A maximum of 59 *S. calcitrans* on traps in this study equates to 0.07 flies per cm^2^ per week. Whilst, for example, a mean of 152.6 flies per day were trapped on blue/alsynite traps, equating to c. 0.35 flies per cm^2^ per week, at a dairy facility in Florida [13]. This is similar to the 0.49 per cm^2^ per week (189 per day) caught on alsynite traps at dairy farms on Reunion Island [20]. However, more comparable numbers of *S. calcitrans* were caught at a zoological park in Virginia, USA, at 0.02–0.16 per cm^2^ per week (mean of 47 to a max of 386) [21]. The experimental farm site in this study acts as a government model farm with high hygiene and manure management standards; the authors were aware that some privately-owned farms had greater *S. calcitrans* populations (A.K. Murchie pers. obs.). However, for the purposes of this study, comparatively low *S. calcitrans* catches may not be a disadvantage; subtle effects on trap efficiency may be masked by very large trap catches and also increasing numbers of flies caught on traps may inhibit other flies from landing [22].

The black frame surrounding the white trap increased *S. calcitrans* catch five-fold, compared to a white or yellow frame. The hypothesis that black–white contrast is attractive to *S. calcitrans* was supported by these data. This is not surprising given the laboratory results of Brady and Shereni [15] and similar results reported for *Musca domestica* [23] whereby contrast was highly attractive to both these nuisance flies. The reasons for this attraction are not fully understood but seem related to conspicuousness. Howard and Wall [23] found that a white square on a black frame caught most *M. domestica* when against a white background (and vice-versa for a black square on a white frame against a black background), concluding that the contrast between the perimeter and the background most affected attraction. In this study, the farmyard sites were largely light grey concrete, breezeblock, and galvanised steel doors, forming a light-coloured backdrop. Attraction to trap contrast against a background may partly explain some of the variability in responses of *S. calcitrans* to colour found across different studies, where background colour was not similar or evaluated. It has been speculated that dark blue is attractive to haematophaghous flies because in the fly’s vision, which is sensitive to ultraviolet light, it provides a contrast with the background green foliage [10]. This emphasises that colours visible to humans may also be producing unknown levels of UV reflection. Agee and Patterson [5] found peak visual sensitivities of *S. calcitrans* to be in the UV and blue green regions of the spectrum.

Attraction to contrast may be of value for *S. calcitrans* in either mating or host location. For the former, male *S. calcitrans* perch on prominent sunlit sites and intercept passing females [24]. In this study, there was no difference in the sex ratios between treatments but this does not discount the possibility of visually conspicuous objects acting as marker sites for mating. There is also the possibility that perching or ‘waiting sites’ aid in thermoregulation with flies basking in the sun to raise core temperature [24]. This could be particularly important in a cool climate such as Northern Ireland. The thermal radiance of the trap could therefore also play a role in attracting alighting *S. calcitrans*.

*Stomoxys calcitrans* are daytime active and use both visual and olfactory cues to host-locate [11,25]. As with other haematophagous Diptera, *S. calcitrans* are attracted to dark silhouettes and possibly to darker—rather than lighter-coloured animals [26]. The attraction of *S. calcitrans* to black-white contrast could be a fortuitous adaptation to black and white Holstein Friesian cattle, which were the main livestock hosts at this locality and the predominant cattle breed on many farms across the region. However, the particular responses to black-white patterning may have a more universal explanation. Brady and Shereni [15] found a similar response to black-white contrast in both *S. calcitrans* and tsetse fly *Glossina morsitans morsitans.* Both species were attracted to a black stripe on a white background, although *G. m. morsitans* were attracted to a horizontal stripe and *S. calcitrans* to vertical. In addition, when the landing positions were analysed, *S. calcitrans* were landing predominantly within the interface edge region between the two colours. Surprisingly, when the number of stripes was increased, whilst correspondingly becoming narrower, fewer *S. calcitrans* alighted on the target area. This unexpected result agrees with the hypothesis of Waage [27] that stripes camouflage the body edge of the host animal. This was supported by experimental evidence of Gibson [28] and more recently with regional analysis of striping prevalence on equids (e.g., zebras) that found greater stripe numbers and definition in areas where biting flies are most active [29]. Whilst these studies demonstrate a protective effect of striping for the host, they nevertheless suggest the importance of colour contrast in host location.

An incidental result of this study was the significant attraction of cluster flies, *Pollenia* sp., to the yellow framed traps. Larval *Pollenia* spp. are parasites of earthworms and emergent adults migrate to overwintering sites during the autumn, whereupon they can cause problems by hibernating in large numbers in peoples’ houses. Prior to this, they are common on a wide variety of flowers feeding on nectar. Other studies have caught *Pollenia* sp. in yellow sticky traps [30,31,32] but there seems to be no other specific testing of their colour preferences available in the literature. Whether the flies were attracted to yellow alone or the yellow–white contrast is not known from this study, but it is likely that the attraction is due to the flower-seeking behaviour of the adults. Lunau [33] reviewed the colour preferences of Diptera and highlighted the preference of flower-visiting flies for yellow and white. However, specific fly species, which tended to have specialised mouthparts, were attracted to shades of red and blue. This might suggest that yellow and white signals a non-specialised nectar or pollen source, which was attractive to *Pollenia* sp. The role of Diptera in pollination is often subsumed by interest in bees. Yet in the isolated Macquarie Island, Diptera are the only pollinators and correspondingly this has selected for different floral signalling, with typically cream-green flowers [34]. As bee and fly colour perception differs in many aspects, it is likely that colour signalling from flowers to facilitate pollination is community, taxa, and even pollinator-species specific.

## 5. Conclusions

This experiment was preliminary and many more colour combinations could be tested; in particular, the contrast of blue-green as perceived in the ultraviolet spectrum. The results of this experiment do not warrant significant changes to existing *S. calcitrans* trap designs. However, they do highlight the importance of perimeter contrast, which could be added to existing designs with minor modifications. In addition, when placing traps, due cognisance should be taken of background colours to maximise the trap efficacy.

## Figures and Tables

**Table 1:** Mean numbers of *S. calcitrans* and *Pollenia* sp. caught on white sticky traps (225 × 400 mm) surrounded by 100 mm frames of black, white or yellow Corriboard® at a total of five sites, comprising three farmyards and two pasture sites, over six weeks. Means and 95% confidence intervals are back-transformed from the GLMM.

Species	Site Habitat	Frame Colour	Mean No. of Flies per Week	Confidence Intervals
*Stomoxys calcitrans*	Farmyard	Black	20.29	(9.98–41.25)
		White	4.13	(1.97–8.67)
		Yellow	3.12	(1.47–6.61)
	Pasture	Black	0.83	(0.29–2.38)
		White	0.17	(0.03–0.85)
		Yellow	0.17	(0.03–0.85)
*Pollenia* sp.	Farmyard	Black	0.17	(0.03–0.87)
		White	0.06	(0.01–0.60)
		Yellow	0.63	(0.17–2.29)
	Pasture	Black	2.17	(0.53–8.92)
		White	0.42	(0.08–2.10)
		Yellow	6.58	(1.66–26.17)

## References

[1] Taylor D.B., Moon R.D., Mark D.R. (2012). Economic impact of stable flies (Diptera: Muscidae) on dairy and beef cattle production. J. Med. Entomol..

[2] Baldacchino F., Muenworn V., Desquesnes M., Desoli F., Charoenviriyaphap T., Duvallet G. (2013). Transmission of pathogens by *Stomoxys* flies (Diptera, Muscidae): A review. Parasite.

[3] Broce A.B. (1988). An improved alsynite trap for stable flies, *Stomoxys calcitrans* (Diptera: Muscidae). J. Med. Entomol..

[4] Williams D.F. (1973). Sticky traps for sampling populations of *Stomoxys calcitrans*. J. Econ. Entomol..

[5] Agee H.R., Patterson R.S. (1983). Spectral sensitivity of stable, face, and horn flies and behavioral responses of stable flies to visual traps (Diptera: Muscidae). Environ. Entomol..

[6] Beresford D.V., Sutcliffe J.F. (2006). Studies on the effectiveness of Coroplast sticky traps for sampling stable flies (Diptera: Muscidae), including a comparison to alsynite. J. Econ. Entomol..

[7] Cilek J.E. (2003). Attraction of colored plasticized corrugated boards to adult stable flies, *Stomoxys calcitrans* (Diptera: Muscidae). Fla. Entomol..

[8] Zhu J.J., Zhang Q.-h., Taylor D.B., Friesen K.A. (2016). Visual and olfactory enhancement of stable fly trapping. Pest Manag. Sci..

[9] Pospisil J., Zdarek J. (1965). On the visual orientation of the stable fly (*Stomoxys calcitrans* L.) to colours. Acta Entomol. Bohemoslov.

[10] Allan S.A., Day J.F., Edman J.D. (1987). Visual ecology of biting flies. Ann. Rev. Entomol..

[11] Gatehouse A., Lewis C. (1973). Host location behaviour of *Stomoxys calcitrans*. Entomol. Exp. Appl..

[12] Cilek J.E. (2002). Attractiveness of beach ball decoys to adult *Stomoxys calcitrans* (Diptera: Muscidae). J. Med. Entomol..

[13] Geden C.J. (2006). Visual targets for capture and management of house flies, *Musca domestica* L.. J. Vector Ecol..

[14] Schofield S. (1998). Responses to electrified targets and daily activity of *Stomoxys* spp. (Diptera: Muscidae) in Zimbabwe. Bull. Entomol. Res..

[15] Brady J., Shereni W. (1988). Landing responses of the tsetse fly *Glossina morsitans morsitans* Westwood and the stable fly *Stomoxys calcitrans* (L.) (Diptera: Glossinidae & Muscidae) to black-and-white patterns: A laboratory study. Bull. Entomol. Res..

[16] Beresford D.V., Sutcliff J.F. (2008). Stable fly *(Stomoxys calcitrans*: Diptera, Muscidae) trap response to changes in effective trap height caused by growing vegetation. J. Vector Ecol..

[17] Solorzano J.A., Gilles J., Bravo O., Vargas C., Gomez-Bonilla Y., Bingham G.V., Taylor D.B. (2015). Biology and trapping of stable flies (Diptera: Muscidae) developing in pineapple residues (*Ananas comosus*) in Costa Rica. J. Insect Sci..

[18] Broce A.B., Hogsette J., Paisley S. (2005). Winter feeding sites of hay in round bales as major developmental sites of *Stomoxys calcitrans* (Diptera: Muscidae) in pastures in spring and summer. J. Econ. Entomol..

[19] Foil L., Hogsette J. (1994). Biology and control of tabanids, stable flies and horn flies. Rev. Sci. Tech. Off. Int. Epizoot..

[20] Gilles J., David J.F., Duvallet G., De La Rocque S., Tillard E. (2007). Efficiency of traps for *Stomoxys calcitrans* and *Stomoxys niger niger* on Reunion Island. Med. Vet. Entomol..

[21] Ose G.A., Hogsette J.A. (2014). Spatial distribution, seasonality and trap preference of stable fly, *Stomoxys calcitrans* L. (Diptera: Muscidae), adults on a 12-hectare zoological park. Zoo Biol..

[22] Beresford D.V., Sutcliffe J.F. (2017). Evidence for sticky-trap avoidance by stable fly, *Stomoxys calcitrans* (Diptera: Muscidae), in response to trapped flies. J. Am. Mosq. Control Assoc..

[23] Howard J.J., Wall R. (1998). Effects of contrast on attraction of the housefly, *Musca domestica*, to visual targets. Med. Vet. Entomol..

[24] Buschman L.L., Patterson R.S. (1981). Assembly, mating, and thermoregulating behavior of stable flies under field conditions. Environ. Entomol..

[25] Schofield S., Torr S.J., Takken W., Knols B.G.J. (2010). Behavioural modalities of ‘non-vector’ biting Diptera: From olfaction to feeding. Olfaction in Vector-Host Interactions. Ecology and Control of Vector-Borne Diseases.

[26] Parr H. (1962). Studies on *Stomoxys calcitrans* (L.) in Uganda, East Africa. II.—Notes on life-history and behaviour. Bull. Entomol. Res..

[27] Waage J.K. (1981). How the zebra got its stripes-biting flies as selective agents in the evolution of zebra coloration. J. Entomol. Soc. South. Afr..

[28] Gibson G. (1992). Do tsetse flies ‘see’ zebras? A field study of the visual response of tsetse to striped targets. Physiol. Entomol..

[29] Caro T., Izzo A., Reiner R.C., Walker H., Stankowich T. (2014). The function of zebra stripes. Nat. Commun..

[30] Aak A., Birkemoe T., Mehl R. (2010). Blowfly (Diptera, Calliphoridae) damage on stockfish in northern Norway: Pest species, damage assessment and the potential of mass trapping. J. Pest Sci..

[31] Goulson D., Hughes W.O.H., Chapman J.W. (1999). Fly populations associated with landfill and composting sites used for household refuse disposal. Bull. Entomol. Res..

[32] Bilaniuk V., Beresford D.V. (2010). Sampling adult blow flies (*Diptera: Calliphoridae*) at pig carcasses with sticky traps: Effects of trap colour, height, and inclination. ‎Can. Soc. Forensic Sci. J..

[33] Lunau K. (2014). Visual ecology of flies with particular reference to colour vision and colour preferences. J. Comp. Physiol. A.

[34] Shrestha M., Lunau K., Dorin A., Schulze B., Bischoff M., Burd M., Dyer A.G. (2016). Floral colours in a world without birds and bees: The plants of Macquarie Island. Plant. Biol..

